# Solubility-consistent force field simulations for aqueous metal carbonate systems using graphical processing units

**DOI:** 10.1098/rsta.2022.0250

**Published:** 2023-07-10

**Authors:** Blake Armstrong, Alessandro Silvestri, Raffaella Demichelis, Paolo Raiteri, Julian D. Gale

**Affiliations:** Curtin Institute for Computation/The Institute for Geoscience Research (TIGeR), School of Molecular and Life Sciences, Curtin University, PO Box U1987, Perth, WA 6845, Australia

**Keywords:** molecular dynamics, force field, alkaline earth carbonates, graphical processing unit

## Abstract

Crystallization of alkaline earth metal carbonates from water is important for biomineralization and environmental geochemistry. Here, large-scale computer simulations are a useful approach to complement experimental studies by providing atomistic insights and even by quantitatively determining the thermodynamics of individual steps. However, this is dependent on the existence of force field models that are sufficiently accurate while being computationally efficient enough to sample complex systems. Here, we introduce a revised force field for aqueous alkaline earth metal carbonates that reproduces both the solubilities of the crystalline anhydrous minerals, as well as the hydration free energies of the ions. The model is also designed to run efficiently on graphical processing units thereby reducing the cost of such simulations. The performance of the revised force field is compared against previous results for important properties relevant to crystallization, including ion-pairing and mineral–water interfacial structure and dynamics.

This article is part of a discussion meeting issue ‘Supercomputing simulations of advanced materials’.

## 1. Introduction

Alkaline metal carbonates are among some of the most abundant minerals on the Earth’s surface. In particular, they are strongly involved in the regulation of acidity and natural sequestration of carbon dioxide in the world’s oceans, as well as being important for direct sequestration of anthropogenic emissions via mineral carbonation. In addition, carbonate formation is a key part of many biomineralization processes. Besides their geochemical significance, undesirable crystallization of metal carbonates can represent a challenge for industrial plant operation through the formation of scale. Therefore, there is ample reason to further our understanding of systems containing aqueous metal ions and carbonate.

There are two particular areas where further detailed knowledge is especially needed. Firstly, there has been intense discussion regarding the nucleation mechanisms of carbonate minerals, with debate regarding whether it is considered classical or if alternative theories [1], such as the pre-nucleation cluster pathway [2], best describe what occurs. Regardless of the nomenclature, it is evident that there is considerable complexity to the nucleation due to the speciation in saturated solutions [3], including the role of bicarbonate versus carbonate [4], liquid–liquid phase separation, and often the formation of amorphous phases prior to the ultimate emergence of the stable bulk crystalline form at ambient conditions [5,6]. Secondly, it is important to understand the properties of the mineral-water interface, which influences the growth and dissolution of the solid, as well as the binding of other ions or molecules present in the environment. In the case of the stable form of calcium carbonate under standard conditions, calcite, the basal (104) surface typically dominates the morphology with large areas of a relatively clean terrace. This has made it possible to determine the interfacial structure via X-ray reflectivity [7,8], leading to the finding that there are at least two layers of ordered water. More generally, there have been substantial advances in imaging both mineral surfaces [9], including defects and interfacial water using atomic force microscopy [10,11].

Despite the considerable advances in experimental techniques for probing the crystallization and interfacial structure of metal carbonates, there are limits to the level of atomic detail that can be directly determined in such complex systems, both in terms of structure and assigning thermodynamics to individual processes. Here, atomistic simulation can play an important role in rationalizing and augmenting the available experimental data. In particular, molecular dynamics simulations are, in principle, capable of providing structural, dynamical and thermodynamic information for many aspects of such processes involving either aqueous ions or the interface between the mineral and water. However, here there are at least three challenges to consider. Firstly, the timescale for the process of interest must lie within the accessible time that can be simulated, which is typically up to a microsecond, though through the use of specialist hardware it is possible to extend this by several orders of magnitude. The case of magnesium ions in water represents an example of where this becomes a problem as ligand exchange occurs on a timescale that is longer than most simulations [12]. In practice, this problem can be overcome through the use of bias-enhanced simulations, provided a suitable collective variable can be found to describe the process of interest. Secondly, the length scale of the process must also be feasible to simulate. While simulation cells can be of the order of tens of nanometres, which is sufficient for many processes, this is not necessarily the case for nucleation phenomena where the critical size, plus the associated volume of saturated solution, may be comparable to this or even exceed it [13]. Due to the low solubility of metal carbonates, this is a particular problem since at saturation this implies only mM concentrations of ions, which means that large volumes must be simulated to have multiple ions present, also leading to further timescale challenges due to the diffusion limitation of ion encounters. Thirdly, there is the question of the accuracy of the energy/force model being used in the simulation. While it is possible to run *ab initio* molecular dynamics for aqueous metal carbonate systems, it is difficult to obtain sufficient statistical data to obtain well-converged rates or thermodynamics, given that many such simulations only sample a few tens of picoseconds. Even if longer times are feasible, more affordable forms of density functional theory, such as generalized gradient approximations, fail to describe carbonate ions in water accurately due to charge-localization limitations [14]. Consequently, in the near term, it is necessary to use force field models to simulate aqueous metal carbonate systems, which also have accuracy limitations that depend on their parameterization. Of course, the hope for the future will be that machine learning trained on high-level quantum mechanical data may offer increased accuracy at an acceptable computational cost [15].

In this study, we attempt to address some of the above challenges in the simulation of aqueous metal carbonate systems by revisiting the choice of the force field in order to try to improve the accuracy, while also maximizing the computational efficiency. There is already a long history of force fields for calcium carbonate (${\mathrm{CaCO}}_{3}$) and to a lesser extent magnesite (${\mathrm{MgCO}}_{3}$). Many of the early models were focused on accurately capturing the structural properties of the bulk solids using either rigid-ion or shell models [16,17]. As the field progressed, there was a shift toward surface properties [18], initially *in vacuo* and ultimately in the presence of water. In addition, interest in ion pairing in solution, as well as the formation of larger clusters, grew. Early extensions of metal carbonate force fields to water tended to combine parameters from a variety of sources. A potential limitation of such force fields was that the overall thermodynamics of the mineral relative to the ions in solution was generally unknown. A focus of our work has been to advocate that for the simulation of aqueous mineral systems, especially those where crystallization is the objective of the study, it is important to accurately calibrate the solubility given by the force field model: in this way, the thermodynamics of the ions in solution relative to the solid-state will be correctly described. Several force fields that are fitted against the solubility have already been published for calcium carbonate [19,20]. Here, we extend this to all of the alkaline earth metal carbonates. Furthermore, the interaction of carbonate with water is modified to provide a better description of the hydration structure, while the hydration free energies of the metal cations are now fitted to reproduce the more exergonic values of Marcus [21], rather than those of David *et al.* [22].

Beyond the changes to the force field described above, which are largely related to its accuracy, we have also revised our earlier models to target improved computational performance in order to extend the time- and length scales that are accessible. Specifically, we make modifications that allow the force field to be used in the OpenMM [23] code with the aim of enabling simulations on graphical processing units (GPUs). Given the trend towards increasing use of parallel arrays of GPUs for peak computing facilities, in order to maximize computational performance while lowering energy costs, the ability to run on such architectures is essential for large-scale simulations in the future. Below we describe in detail the changes required to make this transition, along with the characterization of the force field in comparison to previous results. Finally, the performance of the GPU implementation of the model is contrasted against the previously used message-passing parallel code.

## 2. Methodology

### (a) Molecular dynamics

All molecular dynamics simulations have been run with OpenMM [23] on one GPU using mixed precision with a timestep of 1 fs. The long-range electrostatics were computed using the particle-mesh Ewald method with a precision of $10^{-5}$. The equations of motions were integrated using Langevin dynamics with the LFMiddle discretization [24], apart from those for the calculation of the water residence time, where we used a custom implementation of the Velocity Verlet algorithm and the CSVR [25] thermostat via the OpenMM python API. Constant pressure simulations used a Monte Carlo barostat to equilibrate the cells at 1 atm and the relevant temperature. The cell was constrained to be cubic for the simulations of a single ion-pair in water, while it was fully flexible for the equilibration of the mineral phases. The equilibrated cells at 300 K were then used to create slabs exposing the most commonly found non-polar surfaces of the minerals, (104) for magnesite, calcite and dolomite and (100) for the aragonite-structured phases. The surfaces were then placed in contact with a water slab of approximately the same thickness and the z-direction was equilibrated while the area of the slab was kept fixed.

### (b) Lattice dynamics

All optimizations of crystalline phases at 0 K have been performed with the latest version of the General Utility Lattice Program, GULP 6.1 [26], including the initial derivation of parameters where the relaxed fitting method was used [27]. In order to fit the bulk free energy to match that required to give a correct solubility, the free energy was computed within the quasi-harmonic approximation using equipartition theory and neglecting zero point energy for compatibility with the target use in classical molecular dynamics.

### (c) Free energy methods

#### (i) Ion pairing

Ion-pairing free energies were computed using PLUMED [28] through the OpenMM-PLUMED interface using well-tempered [29] metadynamics with four independent ‘walkers’ [30] for an aggregated simulation time of 280 ns. Two collective variables were used; the cation–carbon distance and the cation by water coordination number, which was computed using the continuous and differentiable switching function

2.1 $$S(d)=\frac{1-{\left(d-d_{0}\right)}^{6}}{1-{\left(d-d_{0}\right)}^{12}},$$

where $d_{0}$ was set to 1.6, 1.9, 2.1 or 2.3 Å for the ${\mathrm{Mg}}^{2+}$, ${\mathrm{Ca}}^{2+}$ ${\mathrm{Sr}}^{2+}$ and ${\mathrm{Ba}}^{2+}$ cations, respectively. These values were chosen to ensure that the decay of the switching function overlaps with the first peak in the cation hydration shell. The Gaussians were deposited every 1 ps with widths of 0.1 Å and 0.1 in the distance and coordination number collective variables, respectively, and an initial height of $2.5\;\mathrm{kJ}\;{\mathrm{mol}}^{-1}$. A bias factor of 5 was used to progressively reduce the height of the Gaussians and ensure convergence of the calculations.

All metadynamics simulations were run in the NVT ensemble at six different temperatures, from 290 K to 340 K every 10 K, using cubic cells of approximately 50 Å in length, which had been previously equilibrated at the corresponding temperature. For each temperature, the potential of mean force (PMF) was calculated from three independent runs and the reported two-dimensional and one-dimensional free energy surfaces represent the average of these three runs.

#### (ii) Ion hydration

Ion hydration free energies have been computed via free energy perturbation [31,32] using Bennett’s acceptance ratio (BAR) technique [33] implemented in Python as Multistate BAR [34]. The electrostatic and non-bonded interactions were turned off incrementally in 24 linearly spaced stages, with each stage consisting of 5 ns of production with the first 200 ps being equilibration. The Lennard–Jones interactions were scaled using a soft core potential [35,36] to prevent numerical instabilities, and an analytical correction was applied to the final hydration free energies to account for their system-size dependence, as described by Hummer *et al.* [37]. All simulations for ion hydration free energies were run in the NVT ensemble at 300 K using a cubic cell approximately  25 Å in length previously equilibrated isotropically at constant pressure and 300 K.

**Table 1.** Parameters for the aqueous alkaline earth carbonate force field developed in this work. The subscripts c and w refer to atom types in carbonate and water, respectively. The potential parameters are specified for either Lennard–Jones (lj) or Buckingham (buck) forms. All the interactions were tapered to zero between 8 and 9 Å, apart from the $O_{w}$-$O_{w}$ which was truncated at 9 Å. This was done to maintain consistency with the original parameters for the SPC/Fw water potential. However, it should be noted that for inhomogeneous systems the long-range tail correction of this force field cannot be applied. The charges for $C_{c}$ and $O_{c}$ are 1.423285 and −1.141095 a.u., respectively, while the cations all carry the formal charge of +2. Charges for water are as per the original SPC/Fw model.

pair	type	ε (eV)	σ (Å)
$O_{w}$-$O_{w}$	lj	0.00674	3.165492
Mg-$O_{w}$	lj	0.001137	2.72
Ca-$O_{w}$	lj	0.00095	3.25
Sr-$O_{w}$	lj	0.000776	3.52
Ba-$O_{w}$	lj	0.000657	3.82
		A (eV)	ρ (Å)	$C_{6}$ (eV ${\text{Å}}^{6}$)
$O_{w}$-$O_{c}$	buck	12534.455133	0.202	0.0
$O_{w}$-$C_{c}$	buck	12534.455133	0.28	0.0
$H_{w}$-$O_{c}$	buck	340.0000	0.217	0.0
$O_{c}$-$O_{c}$	buck	75032.644	0.198913	28.0
Mg-$O_{c}$	buck	3336.5869	0.244667	0.0
Ca-$O_{c}$	buck	8741.2801	0.244960	0.0
Sr-$O_{c}$	buck	3273.9620	0.283096	0.0
Ba-$O_{c}$	buck	15472.601	0.255163	0.0

### (d) Force field

Previously, we have derived a number of variants of a force field for aqueous calcium carbonate systems with progressive improvements. The motivation for the current re-parameterization is twofold: Firstly, the model has been changed to facilitate its use within the OpenMM code and specifically for the efficient use of GPUs. Secondly, while the model was being modified anyway, a re-assessment of the fitting training set was undertaken. More details regarding both of these points are given below.

By default, OpenMM describes the non-bonded interactions of a system using per-atom type values of sigma and epsilon for a Lennard–Jones potential, which are mixed together using standard combination rules. This format can be overruled in favour of custom non-bonded forces which describe the pair-wise interactions between particles using any potential and per-particle parameters through a Python or C++ API. As such, a great deal of ability to customize the input is afforded to the user, which allowed for the previous non-bonded model (primarily used in LAMMPS) to be converted into an OpenMM-accessible force field without compromise. As a note of caution, the parameters described here are in units of eV for energy and Å for distance, whereas OpenMM requires $\mathrm{kJ}\;{\mathrm{mol}}^{-1}$ and nm for energy and distance, respectively, and so values must be converted appropriately.

The main changes made in this work relative to the 2015 force field [20] are:

— The carbonate–water interaction parameters were changed in order to improve the description of the hydration structure of the anion. This included increasing the absolute values of the charges on C and O within carbonate, while the total charge remained constrained to be −2.
— The cation–water interaction parameters were fitted to target the more exothermic hydration free energies of Marcus [21], rather than the values of David *et al.* [22] that were previously used. The resulting values are more consistent with the cation hydration free energies obtained from quantum mechanical methods. It should be noted that even the hydration free energies of Marcus are near the upper bound of literature values [38], depending on the choice of reference proton hydration free energy. To obtain values close to the lower bound is challenging with a rigid-ion model, due to the need for short cation–water distances, and so ideally a polarizable model should be used instead [14]. However, here we ultimately fit the solubility and so any error in the hydration free energy is subsumed into a corresponding shift in the lattice free energy, such that the free energies of the ions in solution with respect to the solid are correct.
— The tapering function was changed from the Mei-Davenport-Fernando (MDF) form [39] to the built-in OpenMM switching function. Although the MDF tapering function can be hard-coded into the energy expressions, it was decided that the effect of changing the tapering function used did not have a significant effect on the outcome, provided the change was made prior to the fitting of the force field. Likewise, the taper itself was changed from the range of 6–9 Å to 8–9 Å due to the smaller $C_{6}$ term in the $O_{c}$-$O_{c}$ Buckingham potential.
— The intramolecular interactions for carbonate were simplified by the removal of the anharmonic bond/angle coupling terms, leaving just the harmonic bond-stretch, angle-bend and out-of-plane contributions. While the more complex model could be implemented as a custom force, the additional anharmonic terms were originally added to improve the vibrational spectrum of carbonate. However, they are less critical for the thermodynamics of crystallization from water and so it is more efficient to remove them here. The final parameters for the revised force field are given in table 1.

## 3. Results and discussion

The revised force field model described above has been benchmarked against a range of important structural and energetic properties that are relevant to an accurate description of the aqueous metal carbonate system from the initial speciation in solution through to the formation of the bulk minerals and their interface with water during growth. Results for each of these aspects are described in the following sections.

### (a) Bulk mineral phases

Crystalline alkaline earth carbonates represent a rich and diverse class of minerals, that span single-metal anhydrous phases through to hydrated and hydroxylated solid solutions. As the primary application of this force field will be the investigation of prenucleation speciation, crystal growth and dissolution of biominerals, it has been fitted against the solubility of the anhydrous single-metal phases that are most stable at the conditions relevant to biomineralization (i.e. calcite, magnesite, strontianite and witherite). Dolomite and aragonite, whose solubilities were not explicitly fitted, are also considered as other relevant anhydrous phases that can occur either in geological settings or as a biomineral. Hydrated and hydroxylated phases, which can appear as precursors to the anhydrous phases due to either kinetic or particle size-dependent thermodynamic factors [19], have not been considered in this initial phase. This is because there are no additional free parameters that can be fitted to such materials once the interaction of the ions with bulk water are determined. Transferability of the model to these phases is probed and discussed below.

Table 2 shows the structural parameters, density and standard free energy of dissolution that determines the solubility of the aforementioned anhydrous phases. Most lattice parameters are overestimated by 1–3%, resulting in lower densities. While the c lattice parameters of the hexagonal phases (magnesite, calcite and dolomite) are either slightly overestimated or slightly underestimated, the c lattice parameters of the orthorhombic phases (aragonite, strontianite and witherite) tend to be significantly underestimated. Relative stabilities of the Mg-Ca phases match the experimental sign and order of magnitude. While calcite is over-stabilized with respect to aragonite ($\Delta G=2.4\;\mathrm{kJ}\;{\mathrm{mol}}^{-1}$ per ${\mathrm{CaCO}}_{3}$ vs the experimental value of $0.8\;\mathrm{kJ}\;{\mathrm{mol}}^{-1}$ [43]), dolomite has a ΔG of formation with respect to calcite and magnesite of $-10.4\;\mathrm{kJ}\;{\mathrm{mol}}^{-1}$ per $\mathrm{CaMg}{\left({\mathrm{CO}}_{3}\right)}_{2}$, a value that falls in between those obtained from synthetic magnesite ($-12.4\;\mathrm{kJ}\;{\mathrm{mol}}^{-1}$) and natural magnesite ($-8.8\;\mathrm{kJ}\;{\mathrm{mol}}^{-1}$) [43].

**Table 2.** Lattice parameters (a,b,c, Å) and densities (ρ, $g\;{\mathrm{cm}}^{-3}$) for the most common anhydrous alkaline earth metal carbonate mineral phases computed with the force field developed in this work at 0 K from lattice dynamics and at 300 K from a 10 ns long MD simulation. The data are reported as the percentage differences with respect to the experimental values. The standard bulk dissolution free energies ($\mathrm{kJ}\;{\mathrm{mol}}^{-1}$) are reported as determined experimentally and computed at the optimized structures within a classical treatment of the phonons.

mineral		a	b	c	ρ	$\Delta G_{\mathrm{diss}}$
magnesite		
	Exp [40]	4.635	4.635	15.019	3.006	+44.5
	@ 0 K	+0.2	+0.2	−0.7	0.3	
	@ 300 K	+0.3	+0.2	−0.1	−0.4	+44.5
calcite		
	Exp [40]	4.988	4.988	17.061	2.713	+48.5
	@ 0 K	+1.2	+1.2	0.0	−2.3	
	@ 300 K	+1.0	+0.9	+0.8	−2.6	+48.4
dolomite		
	Exp [41]	4.807	4.807	16.002	2.869	+48.7
	@ 0 K	+1.0	+1.0	−1.2	−0.8	
	@ 300 K	+1.0	+1.0	−0.6	−1.3	+51.7
aragonite		
	Exp [42]	4.961	7.967	5.740	2.930	+47.7
	@ 0 K	+1.5	+2.7	−3.8	−0.3	
	@ 300 K	+1.8	+3.0	−2.6	−2.1	+46.1
strontianite		
	Exp [42]	5.090	8.358	5.997	3.844	+52.9
	@ 0 K	+1.1	+0.8	−4.5	+2.7	
	@ 300 K	+1.3	+1.3	−3.2	+0.7	+52.9
witherite		
	Exp [42]	5.313	8.896	6.428	4.314	+48.9
	@ 0 K	+0.7	+0.4	−2.1	+1.1	
	@ 300 K	+0.9	+0.5	1.0	−0.4	+48.9

Overall, this model provides a description of the anhydrous phases considered that is accurate enough to simulate relevant properties. While it is possible to find rigid-ion force field parameters that lead to more accurate structures and better mechanical properties, this would come at the expense of large errors in the solubility [19]. In order to describe both the thermodynamics and structure simultaneously with comparable accuracy, it would be necessary to include polarization effects in the force field, making any large-scale simulations far more computationally demanding.

Transferability to other more complex alkaline earth carbonate phases, such as vaterite which has a complex structure [44,45], has not been assessed in this study. However, examination of the hydrated phases of calcium carbonate was attempted, though with less satisfactory results. For example, molecular dynamics simulations at 300 K of monohydrocalcite—the monohydrated calcium carbonate phase—show a high degree of instability due to a phase transition occurring leading to a different structure. Periodic density functional theory calculations performed using the CASTEP code [46] at the PBE-D3 level with a plane-wave basis set (1000 eV cut-off) and well-converged *k*-point sampling were used to verify that the alternative structure is a distinct minimum, but with an internal energy that is $24.0\;\mathrm{kJ}\;{\mathrm{mol}}^{-1}$ higher than for the experimental monohydrocalcite structure. This failure of the force field for this hydrate is likely to be due to the carbonate–water interactions being parameterized for the ions in solution, rather than for a single water molecule in an ordered solid, as well as the water model itself being originally parameterized for liquid water. Again the lack of polarization in the water model will limit its ability to describe both the liquid phase and isolated molecules in a strong local field due to an ionic solid. Based on the above limitation, this model is not recommended for use in simulating alkaline earth carbonate hydrates.

### (b) Ion properties in aqueous solution

Presented in table 3 are the hydration free energies of the metal cations and the carbonate ion. Good agreement is shown between the calculated values and the experimental values, which is by design, given they were used to guide the parameterization of the force field. The changes in hydration free energies between the 2015 force field [20] to those presented here are due to the cation–water interaction parameters being fit against the values of Marcus [21,47] instead of those from David *et al.* [22]. While it might appear that the hydration free energy of carbonate is inferior in the current model to that previously obtained, it is important to note that there is considerable uncertainty regarding this value, given that the doubly charged carbonate anion doesn’t exist in the gas phase and so this quantity is hypothetical.

**Table 3.** Ion hydration free energies ($\mathrm{kJ}\;{\mathrm{mol}}^{-1}$) obtained via free energy perturbation at 300 K. The experimental values are taken from [21,47].

ion	experiment	previous FF [20]	this work
${\mathrm{Mg}}^{2+}$	−1830/−1837	−1766	−1825.4±0.4
${\mathrm{Ca}}^{2+}$	−1505/−1527	−1444	−1498.6±0.4
${\mathrm{Sr}}^{2+}$	−1380/−1398	−1316	−1379.0±0.4
${\mathrm{Ba}}^{2+}$	−1250/−1270	−1185	−1249.9±0.4
${\mathrm{CO}}_{3}^{2-}$	−1315/−1324	−1312	−1251.0±0.8

Table 4 contains the self-diffusion coefficients and water exchange rates computed using the new force field parameters following the same approach as used previously [20,55]. The newly calculated diffusion coefficients are consistently lower and better reproduce the experimental estimates (except for ${\mathrm{Ba}}^{2+}$ in both cases). Similarly, the water exchange rates exhibit a slight variation from the previous force field, though in the direction of the experimental estimates, except for ${\mathrm{Sr}}^{2+}$. In this last case, the experimental exchange rate appears to be unexpectedly slow given the cation size and water coordination number, whereas the computed values exhibit a consistent decrease going down the periodic table, as might be anticipated. For ${\mathrm{Mg}}^{2+}$, the rate of water exchange is too slow to be determined from an unbiased simulation, as expected given the large barrier height previously computed for a similar model [56].

**Table 4.** Self-diffusion coefficient and water exchange rate computed from 50 ns long MD simulations at 300 K with the CSVR thermostat using a 5 nm cubic water box. No water exchange events were observed for ${\mathrm{Mg}}^{2+}$.

	$D_{0}$ ($10^{-5}\;{\mathrm{cm}}^{2}\;s^{-1}$)			τ (ns)		
ion	exp.	prev. FF [20]	this work	exp.	prev. FF [20]	this work
${\mathrm{Mg}}^{2+}$	0.77 [48]	0.86	0.73	1500 [49]	>100	>50
${\mathrm{Ca}}^{2+}$	0.79 [48]	0.95	0.67	1.1–1.6 [50]	0.23	0.4
${\mathrm{Sr}}^{2+}$	0.79 [48]	0.94	0.75	1.0 [51]	0.36	0.3
${\mathrm{Ba}}^{2+}$	0.84 [48]	0.90	0.95	0.2 [52]	0.13	0.2
${\mathrm{CO}}_{3}^{2-}$	0.8 [53]–0.955 [54]	1.0	0.92		0.017	0.02

**Figure 1.**
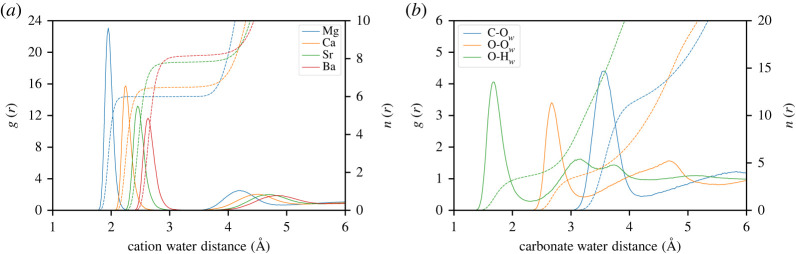
Radial pair distribution functions (left axis) between the alkaline earth metal cations and the oxygen of water (*a*) and between carbonate and water (*b*). Dashed lines represent the integral of the radial pair distribution (right axis). (Online version in colour.)

The ion-water distances in the first hydration shell are presented in table 5 which have been taken from the RDFs shown in figure 1. The average distance between the carbon of carbonate and the oxygen of water in this shell better reproduces the comparable value (3.55 Å) from a polarisable version of this force field based on the AMOEBA model [14], whereas the earlier 2015 variant allowed water closer to carbonate than expected. The distances between the metal ions and the oxygen of water are consistently lower than the experimental values as well as those from the previous force field. As discussed in the previous force field paper [20], the discrepancy with the experimental distances is again due to the lack of polarizability in the force field. The even shorter distances produced by this new force field result from targeting a lower hydration free energy, and with a non-polarizable force field, the only way to achieve this is to reduce the ion-water distance.

**Table 5.** Ion-water distance (Å) in the first hydration shell computed from 50 ns long MD simulations at 300 K with the CSVR thermostat using a 5 nm cubic water box.

ion-$O_{w}$	experiment	previous FF [20]	this work
${\mathrm{Mg}}^{2+}$	2.00–2.15 [57]	2.00	1.95
${\mathrm{Ca}}^{2+}$	2.33–2.44 [57]	2.36	2.25
${\mathrm{Sr}}^{2+}$	2.60–2.65 [57–59]	2.55	2.45
${\mathrm{Ba}}^{2+}$	2.75–2.90 [57–59]	2.75	2.63
${\mathrm{CO}}_{3}^{2-}(C)$	3.35 [60]	3.24	3.57

### (c) Ion pairing

**Figure 2.**
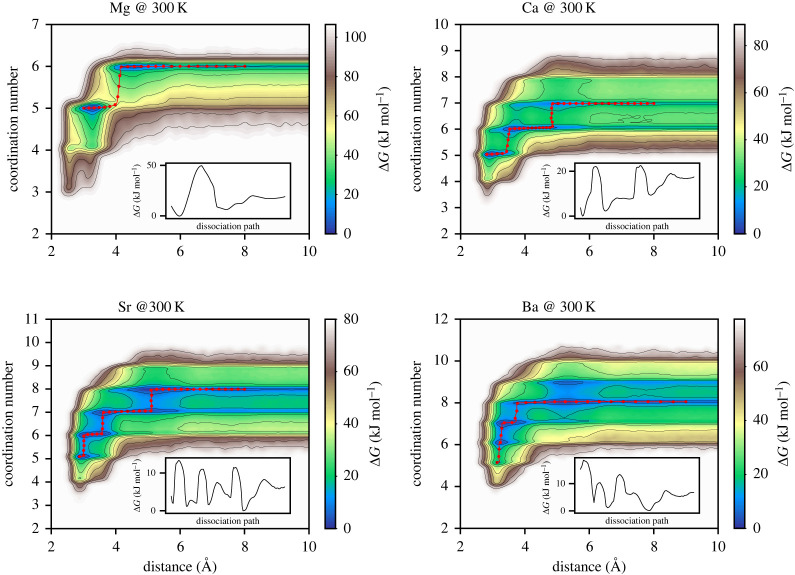
Ion pairing free energy at 300 K as a function of the cation–carbon distance and cation–water coordination number. The minimum free energy paths are shown as red circles overlaid on the two-dimensional free energy map and as one-dimensional profiles in the insets. (Online version in colour.)

The association of calcium and carbonate ions in water prior to nucleation is a topic that has received substantial attention [3,14,61]. Here, ion pairing represents the first step and therefore this process represents an important test for any force field model. Figure 2 shows the two-dimensional maps calculated at 300 K where the free energy of ion pairing is reported as a function of the two metadynamics collective variables employed; the cation–carbon distance and the cation–water coordination number. As shown in our previous study [20], upon reduction of the cation–carbon distance, at a distance of approximately 7 Å, the solvation shells of the two ions come into contact and a solvent-separated ion pair (SSIP) results. When only one water layer separates the two ions, at a distance of approximately 5 Å, a solvent-shared ion pair (SSHIP) is formed. Figure 2 shows that the water coordination number of all the cations remains constant until the SSHIP is formed, suggesting that the carbonate ion is the first species to lose a water molecule when forming the SSHIP. Finally, when the carbonate displaces the shared water molecules, a contact ion pair (CIP) is formed and this can be either mono- or bi-dentate depending on the number of shared water oxygen atoms replaced by the carbonate oxygen atoms. ${\mathrm{Mg}}^{2+}$ can accommodate a maximum of 6 water molecules in its solvation shell and this is reduced to 5 when the CIP is formed. A free energy barrier of $50\;\mathrm{kJ}\;{\mathrm{mol}}^{-1}$ needs to be overcome in order to form the Mg–${\mathrm{CO}}_{3}$ CIP, which is comparable to that for water exchange and twice as large as for the same process with the 2015 force field. This barrier is much lower for all the other metal carbonate ion pairs, because the larger the cation size, the larger its hydration shells, making it easier for the carbonate to replace a shared water molecule. Figure 2 shows how the energetic cost to form the CIP decreases with cation size and this also reflects the decrease of hydration free energy with increasing ionic radius.

**Figure 3.**
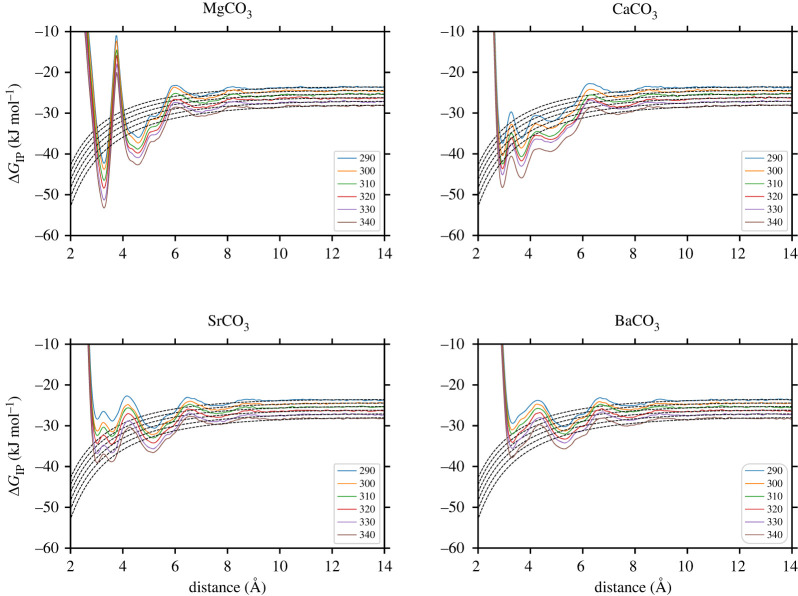
One-dimensional PMF curves for six different temperatures obtained by integrating out the cation–water coordination number collective variable. Dashed lines represent the analytic solution for the pairing free energy of two-point charges interacting via an electrostatic potential that is screened with the dielectric constant of the water model. (Online version in colour.)

**Figure 4.**
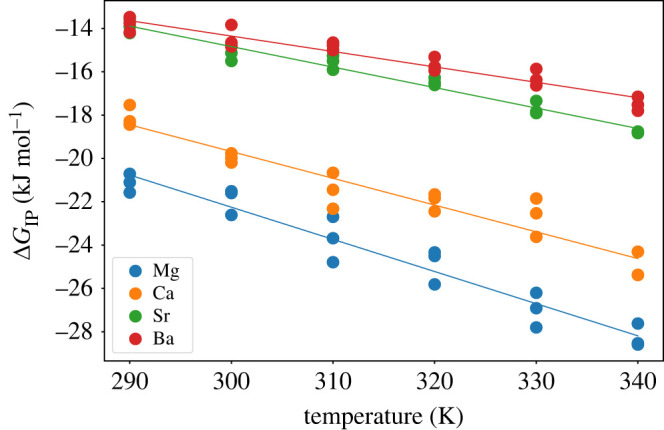
Ion pairing free energy as a function of temperature for the alkaline earth ions and carbonate obtained from integrating the one-dimensional free energy profiles after alignment to the analytic solution. Solid lines represent a linear fit of the data. (Online version in colour.)

Figure 3 shows the one-dimensional PMF curves, ϕ(r), obtained by integrating out the cation–water coordination number collective variable used in the metadynamics simulations. Results are reported for each of the six temperatures studied. The tail of the curves is aligned with the analytic solution for the pairing free energy of two-point charges interacting via a screened electrostatic potential;

3.1 $$\Delta G(r)=\frac{1}{4\pi \epsilon_{0}\epsilon_{r}}\frac{q_{i}q_{j}}{r}-k_{B}T\ln(4\pi r^{2}),$$

where the last term represents the configurational entropy of the system. Once the PMF curves are aligned to the analytic solution, the standard ion pairing association free energy, $\Delta G_{\mathrm{IP}}$, can be computed from;

3.2 $$\Delta G_{\text{IP}}=c^{-o}\int_{0}^{R_{c}}\;e^{-\beta \phi^{\prime }(r)}\;dr,$$

where $c^{-o}$ is a constant necessary to convert the concentration from the simulation units (${\text{Å}}^{-3}$) to standard units ($\mathrm{mol}\;{\mathrm{dm}}^{-3}$) and the aligned PMF curve, $\phi^{\prime }(r)$, is integrated to the end of the bound state. As shown in our previous study [20], the calculated association free energy shows little dependence on the value of $R_{c}$, the upper limit of integration that defines the distance at which the ion pairs can be regarded as dissociated, and here it was set to the Bjerrum length (13.98 Å). For each temperature, $\Delta G_{\mathrm{IP}}$ was calculated from three independent runs and these results are shown in figure 4 together with the corresponding linear fit.

Table 6 reports the experimental and computed ion pairing free energies at 298.15 K, along with the corresponding enthalpy and entropy extracted by fitting the temperature dependence. The computed standard free energies of ion pairing show a trend of decreasing stability as the ions descend the periodic table with most values being within $1.5\;\mathrm{kJ}\;{\mathrm{mol}}^{-1}$ of the experimental estimate. It should be noted that there can be some variation in the experimental value depending on the speciation model used to fit the association data. The exception to the good agreement between calculated and experimental values is for ${\mathrm{Mg}}^{2+}$, where the latter value is less exergonic than for ${\mathrm{Ca}}^{2+}$ and therefore seemingly departs from the size-dependent trend. This is because the contact ion pair becomes less stable than the solvent-shared state due to the strong hydration of the Mg cation. While this departure from the trend cannot be reproduced by a rigid-ion force field, it has been reproduced by a polarisable AMOEBA-based model [56]. In contrast to the free energies, there is substantial scatter among both the experimental and computed enthalpies and entropies making validation difficult. However, the computed enthalpies and entropies at least show a reasonable and consistent trend towards smaller values with increasing cation size.

**Table 6.** Standard free energies, enthalpies and entropies for ion pair association between the alkaline earth cations and the carbonate anion at 298.15 K. Free energies and enthalpies are given in kJ/mol, while entropies are in $J\;K^{-1}\;{\mathrm{mol}}^{-1}$. The uncertainty on the computed ion association enthalpy and entropy are the standard errors given the linear regression of the ion association free energies obtained from the metadynamics simulations that are shown in figure 4.

cation	$\Delta G_{\exp}$	$\Delta H_{\exp}$	$\Delta S_{\exp}$	$\Delta G_{\mathrm{calc}}$	$\Delta H_{\mathrm{calc}}$	$\Delta S_{\mathrm{calc}}$
${\mathrm{Mg}}^{2+}$	−15.6/−16.3	+16.6/+115.2	+108/+106	−22.0	+21.6±3	+146±10
${\mathrm{Ca}}^{2+}$	−19.0/−17.9	+21.4/+8.7	+136/+89	−19.5	+17.4±3	+124±10
${\mathrm{Sr}}^{2+}$	−16.0	+24.8	+137	−14.7	+13.5±1	+93±4
${\mathrm{Ba}}^{2+}$	−15.4	+17.5	+110	−14.2	+7.0±2	+71±5

### (d) Mineral–water interfaces

The structure of the mineral–water interfaces was studied for all the anhydrous phases that have experimentally well-defined non-polar surfaces. For the case of the calcite-structured phases, the (10$\bar{1}$4) surface dominates the morphology and has been extensively studied by techniques including X-ray reflectivity and atomic force microscopy, where atomic resolution can be achieved, including for surface defects, as well as the identification of ordered water layers at the interface. For the aragonite-structured phases, there is less known about the interfacial structure of water.

To simulate the aqueous interface for magnesite, calcite and dolomite, we created a 10-layer thick slab using a 6×3 supercell of the (10$\bar{1}$4) surface unit cell, which was then placed in contact with a water slab of approximately the same size. In the case of aragonite, strontianite and witherite, we created a 10-layer thick slab of a 5×3 supercell of the mineral exposing the neutral (100) surface, which was also placed in contact with a slab of water of equivalent size. All structures were created from unit cells for the bulk mineral which had been previously equilibrated at 300 K with a fully flexible barostat. The analysis of the water density and calculation of the water residence times have been performed using MD simulations in the NVT ensemble that ran for a minimum of 50 ns, after the cell was previously equilibrated in the NPT ensemble at 1 atm, with the slab area kept fixed. In the case of calcite, we extended the simulations up to more than 500 ns in an attempt to determine the water residence time without using enhanced sampling techniques.

**Figure 5.**
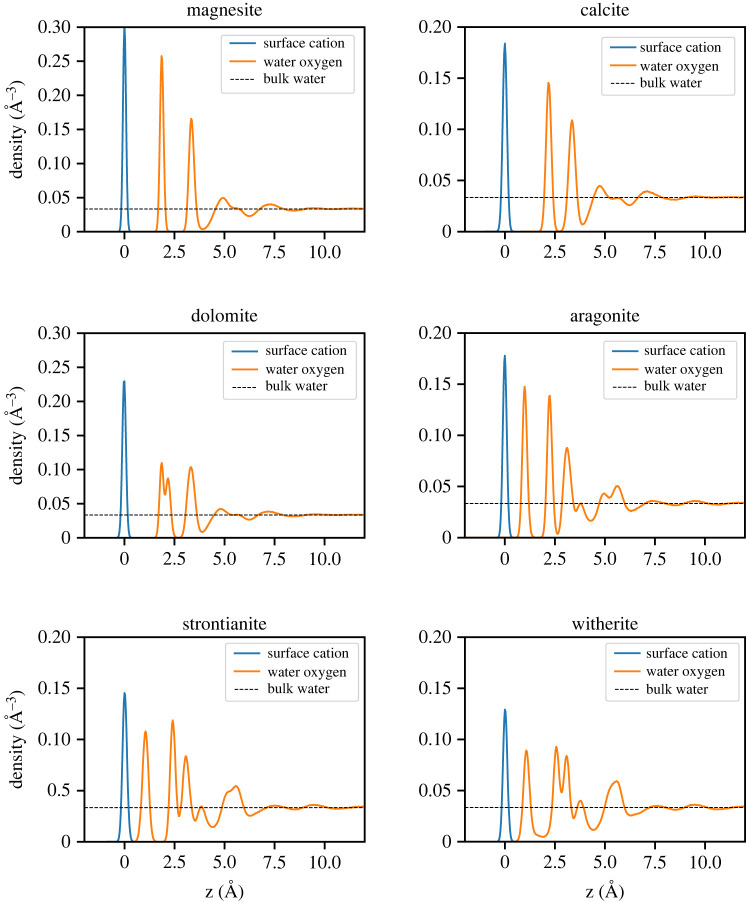
One-dimensional water density profiles normal to surface computed for the mineral-water interfaces of the anhydrous carbonate minerals (orange lines). The density peak (blue) of the cations indicates the position of the surface and the dashed horizontal line is the equilibrium bulk density of water. (Online version in colour.)

All the one-dimensional water density profiles computed in this work (figure 5) show the typical layered interfacial water structure that one would expect for carbonate minerals. In the case of calcite, for which there is ample experimental and computational data to compare against, see e.g. [7,8], the force field developed in this work shows a first water layer, which is formed by molecules located above the calcium ions, at a distance of 2.2 Å from the surface, and a second water layer, which is formed by molecules hydrogen bonded to the surface carbonate anions, at a height of 3.4 Å, both of which are consistent with literature values (1.9–2.36 Å and 3.1–3.5 Å for the first and second water layers, respectively) [7,8]. For magnesite, the first water peak is shifted slightly closer to the surface (1.9 Å) while the second peak remains largely unchanged (3.34 Å), which is consistent with the ${\mathrm{Mg}}^{2+}$ being smaller and more strongly hydrated and the position of the second peak being determined by the carbonate–water interactions. These observations are also consistent with the one-dimensional water density profile above the (10$\bar{1}$4) dolomite surface, where the first peak is split into two, one for the water molecules above ${\mathrm{Mg}}^{2+}$ and ${\mathrm{Ca}}^{2+}$ at 1.9 Å and 2.2 Å, respectively, while again the second peak remains at about 3.3 Å from the surface(figure 5).

**Figure 6.**
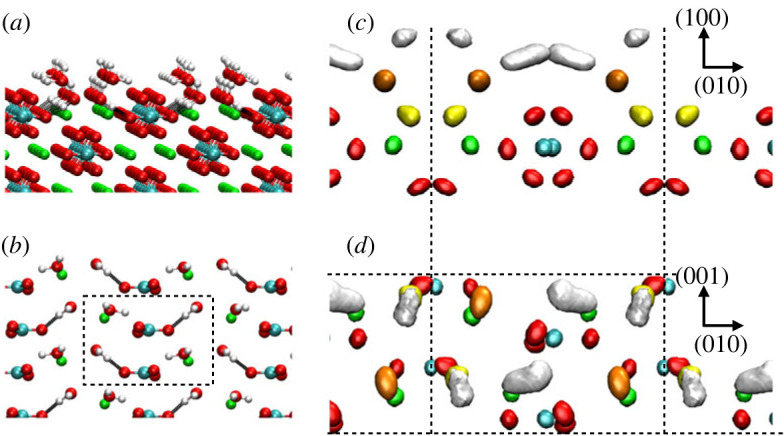
Atomistic representation (*a*,*b*) and three-dimensional density maps of the aragonite-water interface (*c*,*d*). Calcium ions are coloured in green, oxygen atoms in red and hydrogen atoms in white. The same colour code is used for the three-dimensional density maps, apart for those relative to the hydration water, which are coloured in yellow, orange and white to distinguish the first, second and third hydration layers, respectively. In (*a*,*b*), the black lines represent the hydrogen bonds between the water in the first hydration layer and the surface carbonate ions. The iso-density values correspond to approximately 10 times the water bulk density, i.e. $0.3\;\mathrm{atoms}\;{\text{Å}}^{-3}$. The dashed lines represent the boundaries of the surface unit cell and the crystallographic directions are shown on the right-hand side of the figure. For the atomistic representations of the mineral–water interface, the average of 20 successive frames was taken to reduce the distortions in the structure due to vibration. (Online version in colour.)

The water structure above the aragonite-like carbonates is much less studied, both computationally and experimentally. Given the lower symmetry of the orthorhombic structure, there are more distinct surfaces that appear in the morphology of these phases. Here, we focus on the (100) surface as an example, while leaving the comprehensive study of all possible terminations for future work. As found for the calcite basal surface, the aragonite (100) interface also gives rise to substantial ordering of the solvent: Distinct water layers can be clearly identified, with the first one being made up of molecules that are simultaneously interacting with the calcium ions and forming hydrogen bonds with carbonate. The water molecules in the second water layer are predominantly interacting with one surface calcium ion and those in the third layer with a surface carbonate. The same qualitative surface hydration structure is common to all aragonite-like carbonate minerals, with the only differences being the quantitative position and degree of localization of the water molecules, due to the interactions between water and the cations becoming progressively weaker as the cations increase in size (figure 6).

After considering the water structure at the mineral–water interface we turned our attention to the water residence time, which plays a key role in regulating the kinetics of adsorption of ions and molecules at the mineral–water interfaces [62,63]. In particular, a long water residence time, i.e. a slow water exchange rate, requires the coordination of the adsorption site to be explicitly included as a reaction coordinate for the calculation of adsorption free energies using techniques that require the identification of all slow degrees of freedom, such as metadynamics [64] and umbrella sampling [65]. For calcite, dolomite and magnesite we were unable to compute a statistically significant value for the residence time due to the very limited number of water exchange events that were observed over the course of the simulations. In particular, for calcite, we extended the simulation length to almost 600 ns and still only observed a water exchange event for less than 15% of the water molecules in the first hydration layer. It was therefore apparent that advanced sampling methods, such as forward flux sampling [66], would be required to accurately estimate the residence time on these minerals. These results indicate a much longer residence time than that which was previously reported in the case of calcite on its basal plane and at the step edges [55]. While an increase in water residence time is not surprising, given the force field developed in this work has a much more negative hydration free energy for calcium, the magnitude of the increase is greater than might have been anticipated.

In contrast to the situation for the calcite-structure phases, we were successful in computing the water residence time on the aragonite-like mineral surfaces, which was carried out by following the procedure outlined in [55]. From the radial pairing distribution function of the surface cation by water, it was evident that each ion has three neighbouring water molecules in its first coordination shell (within 3.2 Å for Ca and Sr and 3.4 Å for Ba). Hence, the survival function was fitted using the sum of three exponentially decaying functions, one for each of the three different water molecules in the cation coordination shell, table 7. Unlike the cations in aqueous solution, there is no clear trend for the water residence time with size going from ${\mathrm{Ca}}^{2+}$ to ${\mathrm{Ba}}^{2+}$ at this mineral surface. However, the variation by approximately an order of magnitude between the three successive water layers is consistently found for all cations, except for the third layer in the case of ${\mathrm{Ba}}^{2+}$. Taken in combination with the even slower exchange of water at the calcite surface, this illustrates how wide the variation in water exchange rates can be depending on the specific local environment.

**Table 7.** Water residence times for the water molecules in the three hydration layers of the aragonite-like mineral surfaces. All values are in picoseconds.

mineral	water #1	water #2	water #3
aragonite	14	137	2000
strontianite	29	270	2182
witherite	35	260	490

### (e) Performance

**Figure 7.**
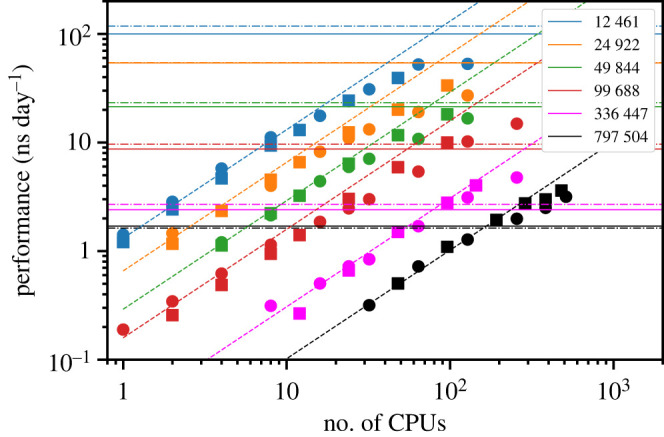
Comparison between the performance of LAMMPS on CPUs (symbols) and OpenMM on GPUs (horizontal lines) at the Pawsey (circles and solid lines) and NCI (squares and dot-dashed lines) supercomputing facilities. The diagonal dashed lines indicate ideal linear scaling. (Online version in colour.)

In addition to the parametrization of a new force field, a key motivation for the present work is to create a model that can be run efficiently on GPUs. Therefore, it is worth briefly discussing the performance of the current force field using this form of hardware within OpenMM. All the calculations reported in this work have been run on the NVIDIA Volta GPU using the CUDA libraries. Figure 7 shows a comparison between OpenMM (GPU) and LAMMPS (CPU) for MD simulations of cubic boxes of SPC/Fw water run on the Pawsey and NCI supercomputing national facilities in Australia. The benchmark simulations were 10 ps long NVT runs with a 1 fs time step for system sizes ranging from 12 k atoms to 790 k atoms, approximately. Although OpenMM can run in parallel on multiple GPUs, we did not see any performance benefit when using more than 1 GPU for any of the system sizes used, so results are only given for the serial case. On the contrary, LAMMPS can run very efficiently across multiple CPUs, and we tested its performance up to 512 cores for the largest system. Our data shows that for small system sizes (less than  50 k atoms) OpenMM on GPUs allows us to obtain more $\mathrm{ns}\;{\mathrm{day}}^{-1}$ of MD than LAMMPS on CPUs before it significantly deviates from linear scaling. On the other hand, the opposite is true for large systems (greater than  100 k atoms) where LAMMPS produces more ns/day of MD than OpenMM when still using large numbers of CPUs efficiently (greater than  100 cores). The choice of which hardware/code to use is therefore a trade-off of multiple factors, which include the Service Unit charging regime given that GPUs often have an additional multiplier per hour of use. Because all simulations performed for this work used relatively small simulation cells, the combination of OpenMM and GPUs offered greater performance. However, it is important to note that the comparison between GPUs and parallel CPUs will very much depend on the specific processors and interconnect available.

It is important to note that LAMMPS also has a GPU package, which can be used to offload some of the calculations onto this form of processor. However, due to the use of hybrid pair styles (Buckingham and Lennard–Jones) in the current force field, we could not achieve good performance of the code using the LAMMPS/GPU combination and found that a CPU-only calculation always outperformed a calculation using one GPU for this part of the calculation. We have also verified for a number of calculation types (single-point calculations, free energy perturbation and an ion pairing free energy) that LAMMPS and OpenMM give consistent results within the statistical noise of the simulations.

## 4. Conclusion

In this study, we have generated a revised rigid-ion force field for aqueous calcium carbonate systems that targets the more widely used and exergonic hydration free energies of Marcus, while aiming to have lattice free energies that are consistent with the solubility for the anhydrous alkaline earth metal carbonates. While qualitatively many of the results of using this new force field are similar to our previous version [20], there are quantitative differences. In order to reproduce the more negative hydration free energies, the metal–water distances are required to be shorter, as seen in the radial distribution functions. Arguably the most notable change is that the new force field leads to significantly longer water residence times at the surface of calcite-structure minerals. Although we have only examined the case of the terraces here, it is likely that the same trend would also apply to steps and kinks. As a result, it will be important to perform bias-enhanced simulations that include a collective variable for the hydration state of the binding site when using this model to study ion adsorption processes at mineral–water interfaces.

Although the new force field developed in this work provides a good description of thermodynamic properties for solubility (by design) and ion pairing in aqueous solution, it is important to recognize that there are limitations. In order to obtain the correct lattice free energy, this means that compromises have to be made in terms of the accuracy of structures and mechanical properties. Indeed, most of the weaknesses of the present force field arise from the lack of a description of polarization, which is necessary to capture the transition between the liquid phase and solid state. While models exist that include this contribution, and consequently show better transferability [14], this comes at the price of significantly increased computational cost. Therefore, the current rigid-ion force field is intended to be used for large-scale molecular dynamics simulations of crystallization processes where other models would prove too costly.

Finally, a feature of the current modified force field is that it is designed to be able to run within the OpenMM code, which has allowed the simulations in this work to be performed on GPUs rather than a parallel architecture. To provide an indication of the performance that is possible, a range of sizes of calcite supercells were run on a single NVIDIA Tesla V100 GPU. For a relatively small system of 5000 atoms, it was feasible to achieve more than 250 ns of simulation per day, though this value decreases rapidly with increasing system size to approximately 10 ns per day for 100 000 atoms. Another way of viewing the performance is that a single GPU is comparable to the order of 100 cores of a supercomputer in parallel (the specific value depends on system size, etc.). This makes the GPU implementation of the present force field particularly efficient in terms of reducing energy consumption while facilitating long simulation times for small systems.

## Data Availability

Files can be obtained from the authors on request.
